# Cadherin-9 Is a Novel Cell Surface Marker for the Heterogeneous Pool of Renal Fibroblasts

**DOI:** 10.1371/journal.pone.0000657

**Published:** 2007-08-01

**Authors:** Cornelia Thedieck, Hubert Kalbacher, Markus Kuczyk, Gerhard A. Müller, Claudia A. Müller, Gerd Klein

**Affiliations:** 1 Section for Transplantation Immunology and Immunohematology, Center for Medical Research, University Medical Clinic, Tübingen, Germany; 2 Medical and Natural Sciences Research Centre, University of Tübingen, Tübingen, Germany; 3 Department of Urology, University Medical Clinic, Tübingen, Germany; 4 Department of Nephrology and Rheumatology, Georg-August-University Medical Center, Göttingen, Germany; L' Istituto di Biomedicina ed Immunologia Molecolare, Consiglio Nazionale delle Ricerche, Italy

## Abstract

**Background:**

Interstitial fibroblasts are a minor, but nevertheless very important, component of the kidney. They secrete and remodel extracellular matrix and they produce active compounds such as erythropoietin. However, studying human renal fibroblasts has been hampered by the lack of appropriate surface markers.

**Methods and Findings:**

The expression of cadherin-9 in various human renal cell lines and tissues was studied on the mRNA level by RT-PCR and on the protein level with the help of newly generated cadherin-9 antibodies. The classical type II cadherin-9, so far only described in the neural system, was identified as a reliable surface marker for renal fibroblasts. Compared to FSP1, a widely-used cytosolic renal fibroblast marker, cadherin-9 showed a more restricted expression pattern in human kidney. Under pathological conditions, cadherin-9 was expressed in the stroma of renal cell carcinoma, but not in the tumor cells themselves, and in renal fibrosis the percentage of cadherin-9-positive cells was clearly elevated 3 to 5 times compared to healthy kidney tissue. Induction of epithelial mesenchymal transition in renal epithelial cells with cyclosporin-A, which causes renal fibrosis as a side effect, induced cadherin-9 expression. Functional studies following siRNA-mediated knockdown of cadherin-9 revealed that it acts in the kidney like a typical classical cadherin. It was found to be associated with catenins and to mediate homophilic but not heterophilic cell interactions.

**Conclusions:**

Cadherin-9 represents a novel and reliable cell surface marker for fibroblasts in healthy and diseased kidneys. Together with the established marker molecules FSP1, CD45 and α smooth muscle actin, cadherin-9 can now be used to differentiate the heterogenic pool of renal fibroblasts into resident and activated fibroblasts, immigrated bone marrow derived fibroblast precursors and cells in different stages of epithelial mesenchymal transition.

## Introduction

The most prominent cell type of the adult kidney is the polarized epithelial cell, and in the healthy organ only a few spindle-shaped fibroblasts can be found in the interstitial space between renal tubules and collecting ducts. Nevertheless, these renal fibroblasts represent a functionally essential part of the kidney. During embryonic development, the epithelial tubules are generated from the metanephric mesenchyme by an inductive process known as MET-mesenchymal to epithelial transition [1], [2]. Embryonic mesenchymal cells which do not undergo MET can form the cells of the renal stroma [3], but the heterogeneous renal stromal cells in the adult kidney, including the interstitial fibroblasts, are not very well characterized. This is mainly due to the fact that unambiguous marker molecules for stromal cells are not readily available. So far, the most intensively characterized renal fibroblast marker is FSP1, fibroblast-specific protein 1 [4], but it has the disadvantage that it might also be expressed by macrophages and monocytes, as currently discussed [5]. Functionally, renal fibroblast-like cells are proposed to play a role in tissue regeneration [6], and are also responsible for extracellular matrix secretion and erythropoietin production [7]–[9]. Under pathological conditions during renal fibrosis, increased numbers of renal fibroblasts can be observed. Tubulo-interstitial fibrosis, which is regarded as the common final pathway of end-stage renal disease, is characterized by the accumulation of extracellular matrix deposited by the renal fibroblasts [10], [11]. Emerging evidence suggests that the disease-related fibroblasts originate from tubular epithelial cells in a process dubbed EMT-epithelial to mesenchymal transition [12]–[14].

Little is known about the role of cadherins in mesenchymal cells of the kidney. The cadherins constitute a large gene family of multifunctional cell adhesion receptors which play pivotal roles not only in embryonic development, but also in the maintenance of tissue architecture [15]–[17]. Cadherins mainly mediate Ca^2+^-dependent homophilic cell-cell interactions, but they can also be involved in signal transduction processes [18], [19]. Based on their structural and functional organization, cadherins are subdivided into different subfamilies, the most intensively studied being the classical type I and II cadherins [20], [21]. In the human kidney, a complex pattern of cadherin expression is seen. Epithelial cells of the proximal tubules express N-cadherin and cadherin-6 (K-cadherin), whereas in distal tubules, E-cadherin and cadherin-16 (Ksp-cadherin) are present [22]–[25]. R-cadherin and cadherin-8 seem to play a role in the differentiating metanephric mesenchyme only during kidney development [26]–[28]. So far, human cadherin-9, a classical type II cadherin, has only been described in the brain [29], but an RT-PCR screening of renal cells and tissues for classical type II cadherins indicated cadherin-9 as a stromal cadherin of the human kidney.

In the present study, therefore, we analyzed in detail the expression pattern of cadherin-9 in the developing and adult human kidney. Expression of cadherin-9 was also studied under pathological conditions in renal fibrosis and renal cell carcinoma, as well as in chemically induced epithelial-mesenchymal transition. Because cadherin-9 was found to be exclusively expressed by renal fibroblasts, its expression pattern was used, in combination with other stromal cell markers, to differentiate between the heterogeneous fibroblast subpopulations in the human kidney. The functional activity of cadherin-9 on renal mesenchymal cells was analyzed in cell adhesion and migration assays.

## Materials and Methods

### Renal cell lines and tissues

All cell lines used were cultured in RPMI-1640 with glutamine and 25 mM HEPES (PAA, Coelbe, Germany) supplemented with 10% FCS. The renal fibroblast cell lines TK 173 and TK 188 as well as the renal epithelial cell line TK 163 have been described earlier [30]. The RCC cell lines used were those described recently [25]. Biopsies of fibrotic kidneys were taken for diagnostic reasons. Fibrotic tissues and fibroblast cell lines with the corresponding histopathology are listed in Table 1. Specimens of RCC tissues and the surrounding, histologically normal kidney tissues were obtained after informed written consent from RCC patients at the time of surgery. In addition, an unaffected normal kidney was also used. All tissue samples were immediately snap frozen in liquid nitrogen. The study was approved by the local ethics committees of the universities of Tübingen and Göttingen. Total RNAs of embryonic kidneys from pregnancy weeks 6, 9 and 12 were received from ViroGen (Watertown, MA).

**Table 1:** Histopathologies of fibrosis patients and the corresponding tissues of renal fibroblast cell lines.

	sex/age*	diagnosis
fibrosis patient A	m/55	not known
fibrosis patient B	f/47	acute glomerulonephritis, interstitial nephritis
fibrosis patient C	m/30	acute interstitial nephritis, glomerulonephritis
fibrosis patient D	m/48	mesangioproliferative glomerulonephritis
fibrosis patient E	m/72	mesangioproliferative glomerulonephritis
cell line TK 173	m/4	nephrotic syndrome, without fibrosis
cell line TK 188	f/55	IgA nephritis, renal fibrosis

* at time of diagnosis

### Reverse transcriptase polymerase chain reaction (RT-PCR)

RNA was isolated using the RNeasy Mini Kit (Qiagen, Hilden, Germany). The FirstChoice^®^ Human Total RNA Survey Panel was obtained from Ambion (Huntingdon, UK). RT-PCR was performed using the SuperScript™ One-Step RT-PCR with Platinum^®^ Taq (Invitrogen, Karlsruhe, Germany). Primers for cadherin-9 were designed according to the sequence NM_016279 (GenBank): cadherin-9 forward 5′-GCAAGCTTCACACTGACCAA, cadherin-9 reverse 5′-ATCGAGGAGGGTTGTTGTTG.

### Antibodies

Cadherin-9 antisera were produced using a peptide representing a cadherin-9 sequence (Swiss-Prot: Q9ULB4) with a high antigenic index. The specificity of the peptide was confirmed by a BLAST search. Two rabbits were immunized. The antiserum of the first rabbit performed well in immunohistochemistry and was affinity purified using the antigenic peptide. The antiserum of the second rabbit was IgG-purified and employed for Western blot analysis. The specificity of both antisera was verified in an ELISA using the antigenic peptide. All other antibodies used are listed in Table 2.

**Table 2:** Sources of the used antibodies

antibody	Source
anti FSP1/S100A4 Ab-8	Dianova (Hamburg, Germany)
anti E-cadherin (clone HECD-1)	TaKaRa (Otsu, Japan)
anti cadherin-6 (clone 2B6)	Acris antibodies (Hiddenhausen, Germany)
anti Ksp-cadherin (clone 4H6/F9)	Zymed Laboratories (San Francisco, CA)
anti α_V_-integrin (clone SAM-2)	Zymed Laboratories
anti β-catenin (clone 14)	BD Transduction Laboratories (Heidelberg, Germany)
anti α-smooth muscle actin (clone 1A4)	Sigma (Taufkirchen, Germany)
anti vimentin (clone V9)	Sigma
anti γ-catenin (clone 15F11)	Sigma
anti CD31 (clone Rb10)	kind gift from Rupert Hallmann (University of Münster, Germany)
anti CD45 (clone TL-1)	Leukocyte Typing IV, Workshop no. N818 (31)
Cy3™-coupled goat anti rabbit	Dianova
FITC-coupled goat anti mouse	Dianova
alkaline phosphatase-conjugated goat anti rabbit	Dianova
alkaline phosphatase-conjugated rabbit anti mouse	DAKO (Hamburg, Germany)

### Immunohistochemistry and immunofluorescence staining

The Envision method (DAKO) was applied to detect cadherin-9 in tissue sections mounted in Entellan (Merck). Immunohistochemical images were taken using a Leitz DM-RBE microscope (Leica) with 20×/0.5 and 40×/0.7 objectives at room temperature and the Leica QWin software. For immunofluorescence staining, 5 µm tissue cryostat sections and cells grown in BD Falcon™ CultureSlides (Heidelberg, Germany) were used. After fixation, non-specific binding sites were blocked with 3.75% BSA in TNC (10 mM Tris pH 7.4, 150 mM NaCl, 2 mM CaCl_2_). The primary antibody diluted in blocking solution was applied for one hour, except E-cadherin which was stained overnight at 4°C. After washing in TNC, sections were incubated with Cy3- or FITC-labeled secondary antibodies diluted in blocking solution for one hour. Cell nuclei were visualized by counterstaining with DAPI. Control staining was performed by omitting the primary antibody. All labeled sections were mounted in Mowiol embedding medium. Fluorescent images were visualized using an inverted microscope (Zeiss Axiophot) with 20×/0.50 and 40×/0.75 objectives at room temperature and captured using AnalySIS DOKU^®^ software (Soft Imaging System Corp.). All images were prepared using PhotoImpact^®^ XL (ULEAD) software.

### Immunoblot analysis and immunoprecipitation

Cells were lysed with RIPA (50 mM Tris pH 7.4, 150 mM NaCl, 1 mM EDTA, 1% Triton X-100, 1% sodium deoxycholate, 0.1% SDS and proteinase inhibitors) and tissues with 0.6% CHAPS in PBS containing protease inhibitors. 8 or 12% minigels (Bio-Rad, Munich, Germany) were run at 40mA per gel. After transfer and blocking with 5% skimmed milk in TBS, the primary antibody diluted in blocking solution was applied for 2 hours at 37°C. After washing with TTBS (TBS with 0.05% Tween 20) the membrane was incubated with the appropriate alkaline phosphatase-conjugated secondary antibody. The blots were developed with BCIP/NBT (5-Bromo-4-chloro-3-indolylphosphate/Nitro blue tetrazolium, Sigma). For immunoprecipitations, antibodies were coupled to Protein-A agarose (Sigma). Simultaneously, non-specifically binding proteins were removed from kidney tissue lysates by pre-clearing. After antibody coupling, the beads were washed with PBS and then incubated for 1 hour at 4°C with the pre-cleared kidney lysate. The pellets were washed, resuspended in sample loading buffer, denatured for 5 minutes at 95°C and used for Western blotting.

### Blocking of cadherin-9 antibody with the antigenic peptide

To prove the specificity of the cadherin-9 antibodies in Western blotting and immunofluorescence staining, the diluted antibody was pre-incubated with 20 µg/ml for Western blotting or 40 µg/ml for immunofluorescence staining of the cadherin-9 peptide-ovalbumin conjugate containing the antigenic peptide. As a positive control, the antiserum was pre-incubated without the antigenic peptide-conjugate.

### Downregulation of cadherin-9 by siRNA treatment

TK 188 cells were transfected with two cadherin-9-specific siRNAs HP from Qiagen. TK 188 cells were trypsinized for 15 minutes and 5×10^5^ cells were seeded in 4 ml medium. 5 nM of each cadherin-9-specific siRNA and 20 µl HiPerfect (Qiagen) in 100 µl serum free medium were incubated for 10 minutes at RT and added drop-wise to the cells. As a negative control, cells were transfected with 10 nM of non-silencing control siRNA. The cells were incubated for 48 hours at 37°C and then used in functional assays. The silencing effect was monitored by Western blotting.

### Induction of EMT by cyclosporin-A

TK 163 cells were seeded in BD Falcon™ CultureSlides and grown for 24 hours. Cells were washed with PBS and incubated without or with 4.2 µM or 42 µM cyclosporin-A (Calbiochem, Darmstadt, Germany) in serum-free culture medium for 48h.

### Aggregation assay

siRNA-treated TK 188 cells were harvested with 0.02% trypsin, 20 mM CaCl_2_ in HBSS. Cells were resuspended in assay buffer (1% BSA, 1 mM CaCl_2_, 0.1 mg/ml DNaseI in HBSS). 1×10^5^ cells/200 µl were transferred to a 48-well plate which was blocked with 2% BSA/PBS. Cell aggregation was performed at 37°C and 225 rpm. Cells were fixed at the beginning (t = 0) and after 20 minutes of aggregation with 2.5% glutaraldehyde. Aggregation was quantified by counting particles in a Neubauer chamber. To obtain statistically significant particle numbers, at least three Neubauer chambers were counted per well. The number of particles at t = 0 was set as 100%.

### Statistical analysis

Experimental data of the aggregation assay were analyzed by a two-sided modified Student's t-test and a p-value of data sets equal or lower than 0.001 was considered to be statistically significant.

## Results

### Cadherin-9 expression in human kidney

**Figure 1:**
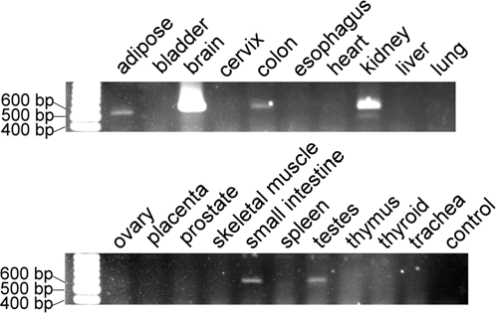
Tissue distribution of cadherin-9. An RT-PCR analysis of a human tissue RNA panel revealed that, apart from the brain, the strongest cadherin-9 expression can be detected in the kidney. Weaker signals could be found in testes (where cadherin-9 was originally described in mice), adipose tissue, colon and small intestine.

**Figure 2:**
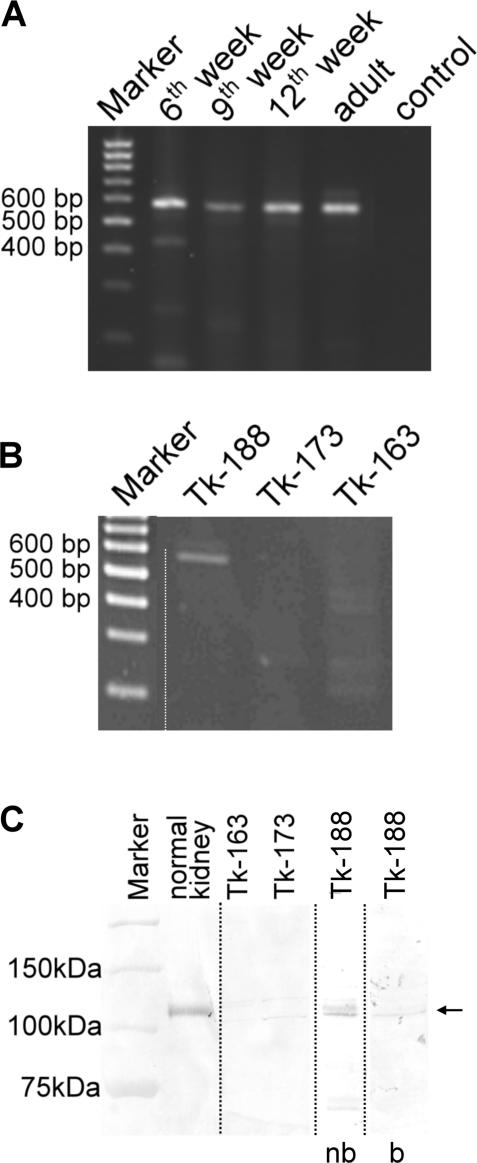
Cadherin-9 appears early in human kidney development and is expressed by a renal fibroblast cell line. (A) During human renal organogenesis, cadherin-9 mRNA is already detectable in the 6^th^ week of gestation. Positive signals were found throughout embryonic development and in the adult kidney. (B) RT-PCR screening of human renal cell lines detected cadherin-9 only in the renal fibroblast line TK 188 which was generated from a fibrotic kidney. Cadherin-9 was neither found in the fibroblast line TK 173 which originates from a normal kidney nor in the renal epithelial cell line TK 163. (C) These results were confirmed by Western blotting with a polyclonal anti-cadherin-9 antibody. The specificity of the anti-cadherin-9 antiserum was shown by pre-incubation of the antiserum with the peptide which the antibody was raised against (nb = non-blocked; b = blocked with peptide).

Initial RT-PCR screening of classical type II cadherin expression in renal tissue had revealed that the mRNA for cadherin-9 was transcribed in the adult human kidney. To study the general tissue distribution of cadherin-9 in humans, a tissue RNA-panel was screened by RT-PCR. In addition to the brain, where human cadherin-9 was originally described [29], the highest expression was observed in kidney. Weaker signals for cadherin-9 expression were found in testes, colon, small intestine and adipose tissue (Fig. 1, Fig. S1). During embryonic kidney development, cadherin-9 expression was already detectable at the 6^th^ week of gestation (Fig. 2A). Strong signals were also obtained at later embryonic stages and in the adult organ. In contrast to human kidney, cadherin-9 was no longer present at the end of embryonic kidney development in the mouse, and was not detectable in the adult kidney (*data not shown*).

To specifically detect cadherin-9 at the protein level, two anti-cadherin-9 antisera were produced. Because cadherin-9 is highly homologous to two other type II cadherins, cadherin-6 and –10, the only cadherin-9-specific peptide sequence with an acceptable antigenic index was found to be located in the cytoplasmic domain from amino acid 701 to 719. A ClustalW alignment of the classical cadherin family confirmed the specificity of the chosen peptide for cadherin-9 (Table 3). Only single amino acids of this peptide are conserved in the other cadherins closely related to cadherin-9. The specificity of the affinity-purified antibodies was verified by blocking with the antigenic peptide using different methods (Fig. 2C, 4A) and by ELISA. A cross reaction of the antisera with the close relatives cadherin-6 or -10 could be excluded, since the antisera did not give positive signals with RCC-cell lines, some of which are positive for the latter cadherins, or cadherin-6-positive renal epithelia.

**Table 3:** ClustalW-alignment of cadherin family members and the cadherin-9 antigenic peptide.

Gene	Sequence	Aminoacid No.
CADH1_HUMAN	R-NDVAPTLMSVPRYL**P**RPANPDEIGN**FI**DENLKAADTDPTAPPYDSLLVFDYEGSGSEA	842
CADH3_HUMAN	LRNDVAPTIIPTPMYR**P**RPANPDEIGN**FI**IENLKAANTDPTAPPYDTLLVFDYEGSGSDA	789
CADH2_HUMAN	RMDERPIHAEPQYPVRSAAPHPG**D**IGD**FI**NEGLKAADNDPTAPPYDSLLVFDYEGSGSTA	867
CADH4_HUMAN	RVDERPVGAEPQYPIR**P**MVPHPG**D**IGD**FI**NEGLRAADNDPTAPPYDSLLVFDYEGSGSTA	877
CADH5_HUMAN	RPSLYAQVQKPP**R**HAPGAHGGPGEMAAM**I**EVKKDEADHDGDGPPYDTLHIYGYEGSESIA	740
CADH6_HUMAN	RRDIVPEALFLP**R**RTPT-ARDNT**DV**RD**FI**NQ**R**LKENDTDPTAPPYDSLATYAYEGTGSVA	747
CADH7_HUMAN	RRDVTPEIQFLS**R**PAFKSIPDNVIFRE**FI**WE**R**LKEADVDPGAPPYDSLQTYAFEGNGSVA	742
CADH8_HUMAN	RKDIKPDLQFMP**R**QGLAPVPNGV**DV**DE**FI**NV**R**LHEADNDPTAPPYDSIQIYGYEGRGSVA	756
CADH9_HUMAN	RRDVMPETIFQI**RRTVP-LWENIDVQDFIHRR**LKENDADPSAPPYDSLATYAYEGNDSIA	747
CAD10_HUMAN	RRDIIPETLFIP**RRT**PT-APD**N**T**DV**R**DFI**NE**R**LKEHDLDPTAPPYDSLATYAYEGNDSIA	745
CAD11_HUMAN	RKDIKPEYQYMP**R**PGLRPAPNSV**DV**DD**FI**NT**R**IQEADNDPTAPPYDSIQIYGYEGRGSVA	752
CAD12_HUMAN	RRDIKPDSLCLP**R**QRPP-MEDNTDIRD**FI**HQ**R**LQENDVDPTAPPIDSLATYAYEGSGSVA	748
CAD13_HUMAN	------------------------------------------------------------	
CAD15_HUMAN	LRRDAPQGRLHPQPPRVLPTSPLDIAD**FI**NDGLEAADSDPSVPPYDTALIYDYEGDGSVA	746
CAD16_HUMAN	GLIVSGPSKDPDLASGHGPYSFTLGPNPTVQ**R**DWRLQTLNGSHAYLTLALHWVEPREHII	742
CAD17_HUMAN	HLFRGPHFTFSLGSGSLQNDWEVSKINGT**H**A**R**LSTRHTDFEERAYVVLIRINDGGRPPLE	755
CAD18_HUMAN	RRDIRPEVKLTP**R**HQTSSTL**E**SIDVQE**FI**KQ**R**LAEADLDPSVPPYDSLQTYAYEGQRSEA	747
CAD19_HUMAN	RKTTSAEIRSLY**R**QSLQVGPDSAIFRKFILEKLEEANTDPCAPPFDSLQTYAFEGTGSLA	730
CAD20_HUMAN	RQDMLPEIESLF**R**YVPQTCAVNSTVHSYVLAKLYEADMDLWTPPFDSLQTYMFEGDGPVA	757
CAD22_HUMAN	QAHLPSERHSLPQGPPSPEPDFSVFRD**FI**S**R**KVALADGDLSVPPYDAFQTYAFEGADSPA	782
CAD23_HUMAN	LLMRGPRPLDRE**R**NSSHV**L**IVEAYNHDLGPM**R**SSVRVIVYVEDINDEAPVFTQQQYSRLG	1217
CAD24_HUMAN	PPARRDVLPRARVSRQ**P**RPPGPADVAQLLAL**R**LREADEDPGVPPYDSVQVYGYEGRGSSC	775
CAD26_HUMAN	LAPVEGRMAETLNQSKERNRFSLSRGCI**I**PQGRATAGRGLPQDIYKEMMPRRLTQTGKRK	765

Bold letters mark the peptide, which was used for the generation of the cadherin-9 specific antiserum and the matching amino acids in the other cadherin-family members. There are only few conserved amino acids, especially in the cadherin-6 and -10 sequences.

Several human kidney cell lines were analyzed for cadherin-9 expression on the mRNA and protein levels. The only cell line found to be positive for cadherin-9 expression was the renal fibroblast cell line TK 188, which was generated from a fibrotic kidney. In contrast, the renal fibroblast cell line TK 173, derived from a non-fibrotic kidney, and the renal epithelial cell line TK 163 were negative for cadherin-9 expression (Fig. 2B). Endothelial cells (e.g., HUVEC) do not express cadherin-9 (*data not shown*).

**Figure 3:**
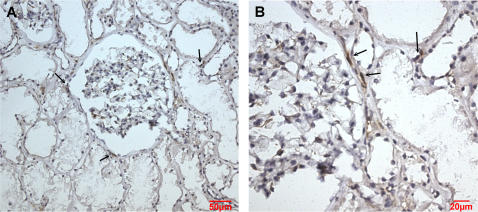
Renal interstitial cells located between tubuli or at the border of glomeruli express cadherin-9. Cadherin-9-positive cells were identified by immunohistochemical staining with the affinity-purified anti-cadherin-9 polyclonal antibody. The sections were counterstained with hematoxylin-eosin. Arrows point to some of the cadherin-9 expressing cells. Panel B shows a part of A in a higher magnification. Bars: (A) 50 µm, (B) 20 µm.

In normal human kidneys, cadherin-9 expression was found to be restricted to single interstitial cells located between renal tubules or outlining the border of the Bowman's capsule. No expression was detectable in tubular epithelial cells or in glomeruli (Fig. 3). These results suggest that cadherin-9 might be exclusively expressed by renal fibroblasts.

### Cellular localization and function

**Figure 4:**
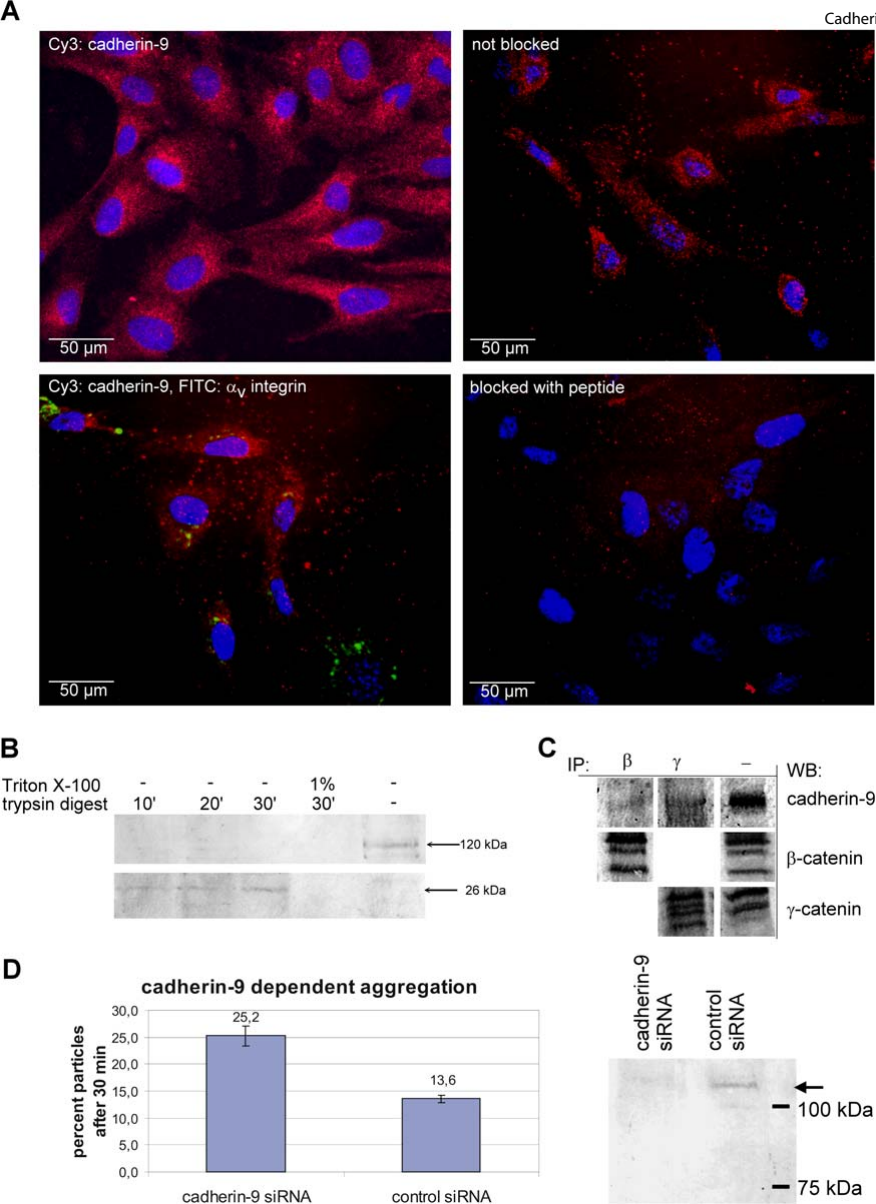
Cellular localization and functional involvement in fibroblast cell aggregation. (A) Immunofluorescence staining of cadherin-9 labeled the whole cell surface of TK 188 cells, and not, as initially expected, only cell-cell contacts. The specificity of the staining was shown by blocking with the antigenic peptide. In double staining experiments, the integrin α_v_-chain which marks focal adhesions showed no co-localization with cadherin-9. Bars, 50 µm. The pictures of the blocking experiment and the double-staining were gamma-corrected (B) To verify the expression on the cell surface, cellular cadherins were digested with trypsin in the presence of EDTA and subjected to Western blotting. After 10 minutes already, no full length cadherin-9 product was detectable, whereas a digestion fragment of approximately 26 kDa appeared. This fragment is most likely the C-terminal end containing the cytoplasmic tail, the transmembrane domain and a radically truncated extracellular domain. This fragment is protected from digestion in the absence of Triton X-100 and can be recognized by the antibody. In the presence of Triton X-100 no cadherin-9 signals were visible. The control cells which were not trypsin-treated showed the full length cadherin-9 product. (C) Co-immunoprecipitation experiments demonstrated the interaction of cadherin-9 with β- and γ-catenin. The pictures of the different immunoblots were gamma-corrected. (D) Cadherin-9 was knocked-down in TK 188 cells by siRNA, and the efficiency of the down-regulation after two days of incubation was shown by Western blotting (on the right). The siRNA treated cells were used for an aggregation assay. The diagram summarizes the data collected in four independent experiments, each of them showing similar results. The capacity to form aggregates was significantly diminished (p<0.00001) in cadherin-9 siRNA-treated cells, since these cells showed, after 30 minutes of aggregation, only 25.2% of their initial number of particles, whereas control siRNA-treated cells showed 13.6%. Error bars indicate the error of the mean.

At the cellular level, cadherin-9 expression was quite evenly distributed over the entire cell surface of the renal fibroblast cell line TK 188 (Fig. 4A). It does not seem to be concentrated at cell-cell contacts nor in focal adhesions which were marked by α_V_ integrin. After fixation with acetone, we could not exclude with certainty that cadherin-9 might also be located in the cytoplasm. Therefore, TK 188 cells were treated with trypsin in the presence of EDTA, which is able to degrade cadherins (Fig. 4B). Already after 10 minutes of trypsin digest, the intact full length cadherin-9 disappeared in Western blots, demonstrating its localization on the cell surface. At the same time a fragment of approximately 26 kDa appeared, which most likely represented the C-terminal part of cadherin-9 including the cytosolic domain, a part of the molecule not accessible to trypsin digestion. After permeabilization of the cell membrane with Triton X-100, the 26 kDa fragment was no longer detectable.

Co-immunoprecipitation of cadherin-9 with β- and γ-catenin in normal human kidney lysates was performed to analyze direct interactions of cadherin-9 with cytosolic catenins, a typical feature of type I and II cadherins. After precipitation with β- or γ-catenin-specific antibodies, cadherin-9 was detectable in Western blot analysis, demonstrating its physical association with both catenins (Fig. 4C).

Cadherin-9 seems to play a role in cell-cell interaction as shown by a cell aggregation assay (Fig. 4D). Cadherin-9 expression was down-regulated in TK 188 cells by siRNA treatment. The efficiency of the siRNA knockdown was evaluated by Western blot analysis. Cadherin-9 expression was completely abrogated after treatment with the specific siRNA. Compared to cells treated with control siRNA, the ability of the cadherin-9-deficient cells to aggregate was significantly impaired (Fig. 4D, Fig. S2). In transmigration and wound closure experiments, however, knockdown of cadherin-9 had no significant effects (*data not shown*). Therefore, cadherin-9 does not seem to influence cell migration, but, despite its atypical distribution over the entire cell surface, nonetheless displays typical features of classical cadherins, namely, interactions with cytosolic catenins and involvement in cell-cell aggregation.

### Cadherin-9 expression is not locally associated with E- or Ksp-cadherins or cadherin-6

**Figure 5:**
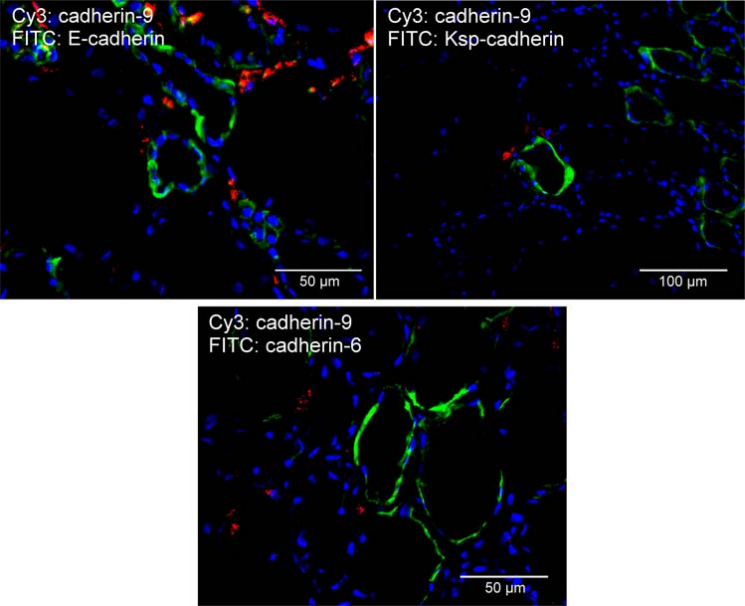
Localization of cadherin-9-positive cells in comparison to other cadherin expressing epithelial tubular cells. The micrographs show double immunofluorescence staining of a normal human kidney with the cadherin-9 antiserum and monoclonal antibodies against E-cadherin (A), Ksp-cadherin (B) and cadherin-6 (C), respectively. E-cadherin and Ksp-cadherin are marker molecules for distal tubules, whereas cadherin-6 is specifically expressed in proximal tubules. However, no preferential distribution of cadherin-9 expressing cells was detected with either proximal or distal tubular epithelial cells, nor any co-localization with the potential interaction partner cadherin-6 was observed. Bars: (A,C) 50 µm; (B) 100 µm.

Because the cadherin family shows a complex expression pattern in the human kidney, the localization of cadherin-9 in relation to the epithelial E- and Ksp-cadherins and cadherin-6 was analysed by double immunofluorescence staining. In an *in vitro* assay, cadherin-9 mediated homophilic interactions, but it could also bind to cadherin-6 and -10 in a heterophilic manner [29]. Cadherin-6 but not -10 is expressed in the adult human kidney (*data not shown*). Double labeling with cadherin-6, which is expressed by proximal tubular epithelial cells, did not reveal any obvious co-localization (Fig. 5), suggesting that *in vivo* in the kidney tissue cadherin-9 does not interact with cadherin-6, although they are able to bind to each other *in vitro*. Kidney specimens were also stained for E- or Ksp-cadherin, which both mark the distal tubules of the human kidney (Fig. 5). Single cadherin-9-positive cells were localized between the distal tubules. No obvious preference for cadherin-9 expression near proximal or distal tubules was observed.

### Cadherin-9 in diseased kidneys

**Figure 6:**
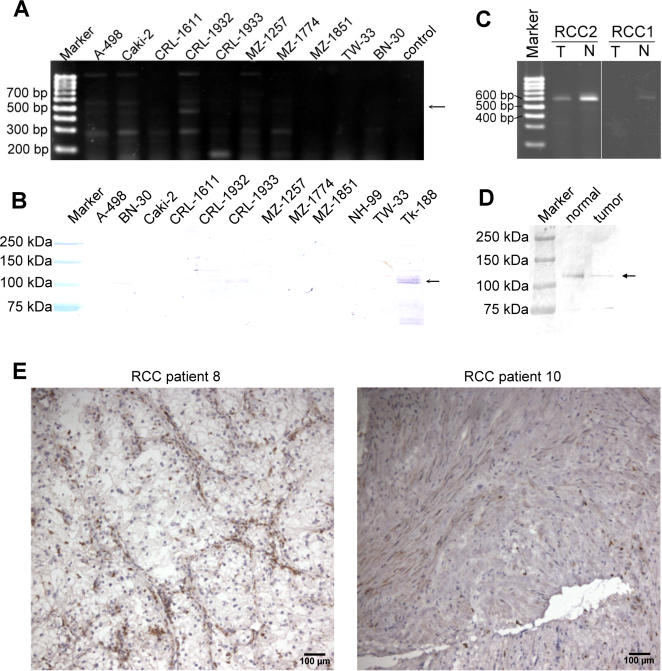
In renal cell carcinoma, cadherin-9 is exclusively expressed by the tumour stroma. (A, B) RT-PCR and Western blot analyses of ten or eleven RCC cell lines, respectively, revealed that cadherin-9 was not expressed in any of the cell lines tested. In the Western blot analysis, the signal detected in the TK 188 cell lysate served as a positive control. (C, D) The signals for cadherin-9 expression detected by RT-PCR or Western blot analyses in renal tumor tissue were constantly much weaker compared to the normal kidney tissue adjacent to the tumor which appeared normal in histology. (E) The immunohistochemical staining of RCC sections showed that cadherin-9 marked exclusively the tumor stroma. Bars, 100 µm. Pictures of the immunoblots (B,D) were gamma-corrected.

The expression pattern of cadherin-9 was also studied under two pathological conditions, renal cell carcinoma and renal fibrosis. In renal tumor tissue, the amount of cadherin-9, compared to the corresponding normal kidney tissue, is considerably lower as shown by RT-PCR and Western blot analysis (Fig. 6C,D). All RCC cell lines tested were negative for cadherin-9 on the mRNA and protein levels (Fig. 6A,B). This result is a strong indication that in renal tumor tissues cadherin-9 is also not expressed by the tumor cells, which are of epithelial origin, but by the stromal cells within the tumor. Immunohistochemical staining of several tumor tissues confirmed this notion. Cadherin-9-positive cells in renal cell carcinomas display an elongated and even spindle-shaped stromal cell phenotype (Fig. 6E). Thus, as in healthy kidneys, cadherin-9 in renal tumors is restricted to stromal cells and cannot be found on epithelial cells.

**Figure 7:**
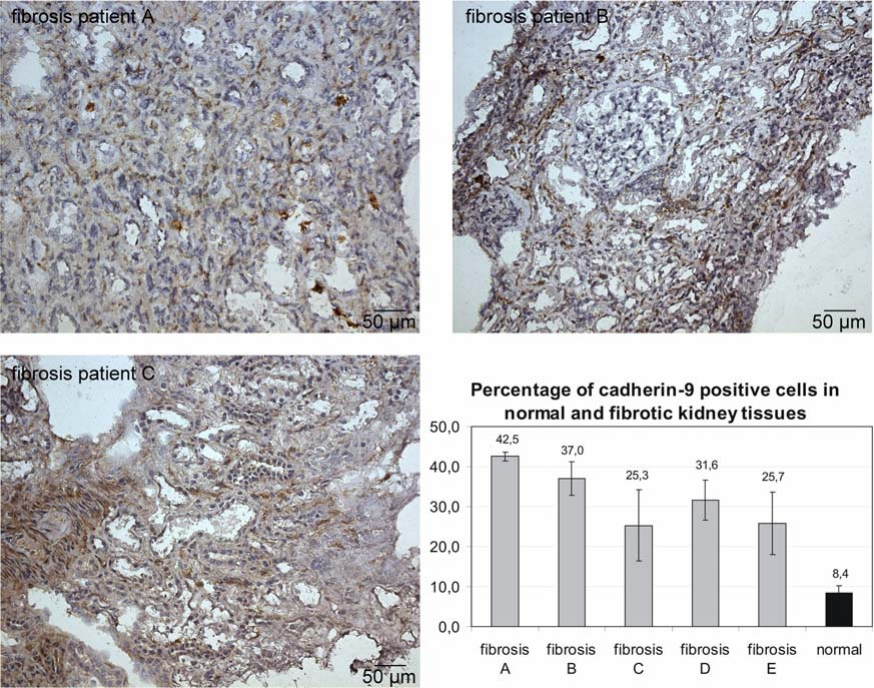
Expression of cadherin-9 in renal fibrosis. Immunohistochemical staining of kidney sections of three fibrosis patients labeled with the cadherin-9 antiserum are shown. The sections were counterstained with hematoxylin-eosin. Cadherin-9-positive cells as well as the total cell number in selected view fields were counted by three independent individuals. Compared to normal kidney sections, a much higher number of cadherin-9 expressing cells could be detected in the fibrotic kidneys. Whereas in normal kidneys only 8.4%±1.8 were positive for cadherin-9 expression, the number of cadherin-9-positive cells in fibrotic kidneys was in the range of 25.7%±7.8 and 42.5%±1.1. Bars, 50 µm.

In renal fibrotic tissues the amount of cadherin-9 positive cells seems to be higher compared to normal kidneys (Fig. 7 and Fig. 3). Expression was quantified independently by three different investigators, who counted total cell numbers and also the cadherin-9 positive cells in representative microscopic view fields. The percentage of cadherin-9-expressing cells was three to five times higher in kidney specimens from fibrosis patients than in healthy kidneys. In fibrotic renal tissue, up to 42.5%±1.1 of the cells displayed cadherin-9, whereas in a healthy kidney only 8.4%±1.8 of the cells expressed this molecule. Because the number of fibroblasts can be greatly elevated in fibrotic tissues, this finding substantiated our theory that the cell type expressing cadherin-9 in the human kidney is indeed the renal fibroblast.

### Presence of cadherin-9 on defined subpopulations of renal fibroblasts

**Figure 8:**
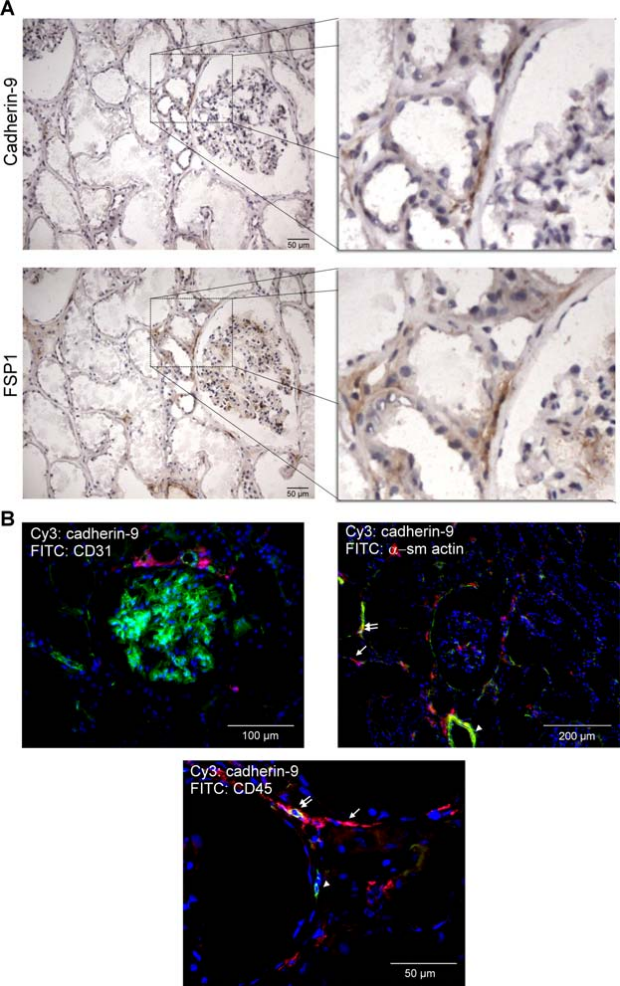
Cadherin-9 is a marker for renal fibroblasts. (A) Immunohistochemical staining of two consecutive kidney cryostat sections with antisera against cadherin-9 and FSP1, a well-known renal fibroblast marker, showed that cadherin-9 positive cells also express FSP1. Staining of FSP1, however, could be also observed in the cadherin-9 negative glomerulum and in some tubular cells. The inserts in the left panel are enlarged in the right panel. (B) Cadherin-9 is not expressed by endothelial cells, as demonstrated by double immunofluorescence staining with the endothelial cell marker CD31. But cadherin-9 expression partially overlaps with CD45 as well as with α-smooth muscle actin. Cells which express only α-smooth muscle actin or CD45 are marked with an arrowhead, whereas cells which express only cadherin-9 with an arrow. Co-localization of α-smooth muscle actin or CD45 with cadherin-9 are indicated by double arrows. The different magnifications in (A) and (B) are shown by different bars.

FSP1 is one of the best described markers for human renal fibroblasts [4]. Immunohistochemical staining of consecutive sections of human normal kidney specimens revealed that cadherin-9-positive cells also expressed FSP1 (Fig. 8A), although there were many more FSP1-positive than cadherin-9-positive cells. These FSP1-positive cadherin-9-negative cells were observed in the interstitium, in glomeruli and intercalating within the tubules. Thus, cadherin-9 expression seems to be more restricted than FSP1.

Cadherin-9 was not expressed by endothelial cells as demonstrated by double staining with the endothelial cell marker CD31 (Fig. 8B). However, some of the cadherin-9-positive cells also expressed α−smooth muscle actin, a marker for smooth muscle cells and activated fibroblasts (Fig. 8B). Because cadherin-9 was not found on smooth muscle cells surrounding large blood vessels and there were also cadherin-9-positive, but α−smooth muscle actin- negative cells present, it seems possible that cadherin-9 is also, but not only, expressed by activated fibroblasts. Some cadherin-9-positive cells co-expressing the leukocyte marker CD45 were also observed (Fig. 8B), but most of the CD45-positive cells did not co-express cadherin-9. Thus, cadherin-9+ CD45+ cells might be immigrated fibrocytes, bone marrow-derived circulating fibroblast precursors, which are known to express CD45 [32].

### Induction of cadherin-9 expression during cyclosporin-A provoked EMT

**Figure 9:**
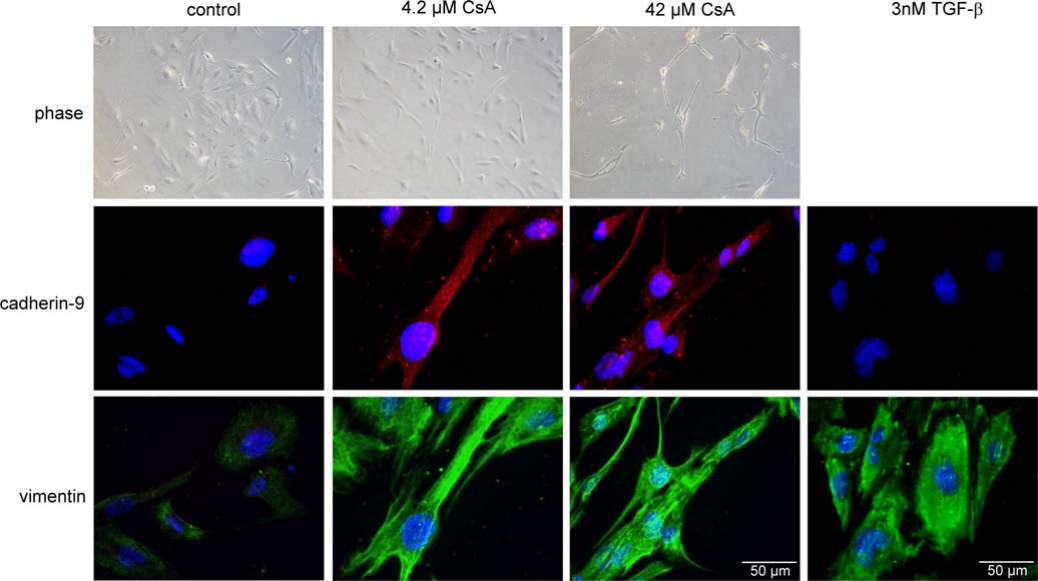
Up-regulation of cadherin-9 expression during cyclosporin-A induced EMT. To induce a transition of renal epithelial cells into a spindle-shaped mesenchymal cell type, the epithelial TK 163 cells were cultured for 48 hours in different concentrations (0, 4.2 and 42 µM) of cyclosporin-A (CsA). EMT could be observed by phase contrast microscopy. Immunofluorescence staining revealed that without CsA no signals for cadherin-9 and only weak signal for vimentin could be observed. Induction of EMT by CsA led to a strong up-regulation of both vimentin and cadherin-9 expression. In contrast, incubation of TK 163 cells with 3 nM TGF-ß for two days does not lead to an induction of cadherin-9 expression. Bar, 50 µm.

A common clinical complication of the immunosuppressive agent cyclosporin-A is a severe tubulo-interstitial fibrosis. One mechanism in developing fibrosis is epithelial-mesenchymal transition of renal tubular epithelial cells **(EMT)**, which can also be induced *in vitro* by cyclosporin-A [33]. Treatment of the renal epithelial cell line TK 163 with cyclosporin-A induced a fibroblastic phenotype. The cells lost their epithelial polygonal shape and gained an elongated polygonal appearance (Fig. 9). This effect was slight at 4.2 µM cyclosporin-A and severe at 42 µM, but the latter concentration was also toxic. Cyclosporin-A-treated TK 163 cells clearly up-regulated vimentin and also started to express cadherin-9 (Fig. 9). Treatment of TK 163 cells with TGF-β1, which is also able to induce EMT but not transdifferentiation into myofibroblasts [34], did not induce cadherin-9 expression. Thus, cadherin-9 up-regulation by cyclosporin-A might be a relatively late event during EMT of renal tubular cells in renal fibrosis.

## Discussion

Here, we identify the classical type II cadherin-9 as a specific and reliable marker molecule for renal fibroblasts. In humans, but not mice, cadherin-9 was found to be present throughout renal development as well as in the adult organ. In renal cell carcinomas its expression is restricted to the stromal compartment of the tumor. Cadherin-9 has the typical homophilic cell adhesion properties of the classical cadherins, whereas heterophilic interactions with epithelial cadherins of the kidney were not observed. Induction of EMT with cyclosporin-A, which can trigger fibrosis in patients, led to an up-regulation of cadherin-9 in converting renal epithelial cells. Consistent with this observation, the percentage of cadherin-9-expressing cells is clearly elevated in renal fibrotic tissue.

In humans, cadherin-9 is mainly expressed in the brain, but it can also be found in kidney, colon, small intestine, adipose tissues and testes. Thus far, cadherin-9 expression had only been described in human brain [29], in the limbic system of rats [35], and in testes and CD4/CD8-double-positive thymocytes of mice [36], [37]. Thus, this is the first description of cadherin-9 in human kidney, intestine and adipose tissue. In contrast to mice, cadherin-9 is not expressed in the human thymus. Additionally, mice lose cadherin-9 expression in the kidney during embryonic development, whereas in the human kidney cadherin-9 is constantly expressed during nephrogenesis and in the adult organ. Mice are therefore not a suitable model to study renal cadherin-9 expression after birth.

In the human kidney, cadherin-9 is exclusively expressed by renal fibroblasts, as shown by the analysis of several renal cell lines and tissues. Cadherin-9 is neither expressed by renal epithelial cells nor by any of the renal cell carcinoma cell lines examined, which were all of epithelial origin. The renal fibroblast cell line TK 188, derived from a fibrotic kidney, is the only cadherin-9-positive cell line found so far. Another renal fibroblast cell line, TK 173, which was established from a non-fibrotic kidney, does not express cadherin-9. TK 188 was found to synthesize higher amounts of extracellular matrix and cytokines compared to TK 173 [38], [39]. Thus, cadherin-9 might have a role in fibroblast activation, as during development of renal fibrosis activated fibroblasts are regarded as responsible for the massive deposition of extracellular matrix, a hallmark of this pathology.

Thus far, there is no commonly-accepted marker molecule for fibroblasts in the kidney. Cadherin-9 might be a good candidate to fill this gap. Currently, the best-studied marker for renal fibroblasts is FSP1, also known as S100A4, pEL98 or mts1 [4], [40]. However, the usefulness of FSP1 as a fibroblast marker is controversial [5]. Some investigators use FSP1 to differentiate fibroblasts from macrophages in the fibrotic mouse kidney [41], while others have reported FSP1 expression on monocytes and macrophages [42], [43]. One possible explanation for this discrepancy might be species differences in the expression pattern of FSP1 [44]. In the human kidney, the use of cadherin-9 as a fibroblast marker will avoid the problems of FSP1, as it is a more restricted marker of renal fibroblasts than FSP1. Compared to FSP1, cadherin-9 antibodies stain fewer cells in the human kidney. Importantly, it is not expressed by any epithelial cells, while FSP1 can be found on single tubular epithelial cells, which might be in the early process of EMT [4]. Cadherin-9 was not expressed by renal tumor cells or leukocytes, as clearly demonstrated in RCC tissues which showed cadherin-9 expression only in the stromal compartment, but not in tumor cells or leukocyte infiltrates. Also the tissue distribution of cadherin-9, which is only expressed in a few organs, seems to be more restricted than FSP1 [40]. Another advantage of cadherin-9, compared to the cytosolic FSP1, is its surface expression which qualifies cadherin-9 as a potential surface marker to sort cells without permeabilization and thus killing the cells.

**Figure 10:**
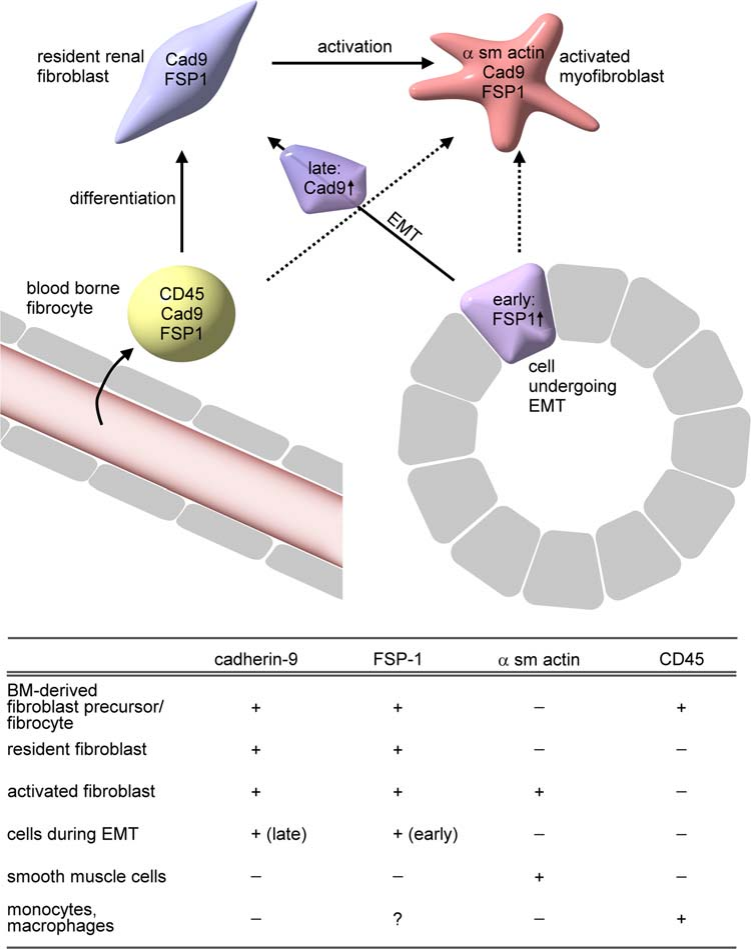
Model of antigen expression in different fibroblast pools, smooth muscle cells and monocytes in the human kidney. Together with FSP1, α-smooth muscle actin (α sm actin) and CD45, cadherin-9 is a novel cell surface marker, which can be used to differentiate the depicted renal fibroblast subpopulations from other renal cell types such as epithelial and endothelial cells, smooth muscle cells, monocytes or macrophages.

In a few cells, co-expression of cadherin-9 and α-smooth muscle actin was found, which might represent activated fibroblasts, as well as co-expression with CD45. The latter cell population could be bone marrow-derived circulating fibrocytes [32], which infiltrated the kidney. Summarizing our data on the different marker molecules, we propose the following model of different fibroblast subpopulations in the human kidney (Fig. 10): Cadherin-9 is expressed by all fibroblast subpopulations of the human kidney defined in this study. In contrast to other markers, it clearly differentiates these fibroblasts from other renal cell types such as epithelial cells, macrophages or smooth muscle cells. The cadherin-9-positive cell population can be subdivided according to co-expression of FSP1, CD45 and α-smooth muscle actin. Fibrocytes co-express the common leukocyte antigen CD45 and FSP1, whereas resident fibroblasts only co-express FSP1. During EMT, FSP1 is up-regulated at an early stage [45], and it can already be found on single tubular epithelial cells, still integrated in the epithelial layer of the tubules. Cadherin-9 appears later during EMT and is undetectable in tubular cells. We found that cadherin-9 expression could be induced by cyclosporin-A, but not by the cytokine TGF-β. Cyclosporin-A, in contrast to TGF-β, not only induces EMT but also myofibroblast transdifferentiation [33], [34]. Calcineurin is the target of Cyclosporin-A in the cell. Thus, the inducibility of cadherin-9 by Cyclosporin-A but not by TGF-β suggests that cadherin-9 transcription might be regulated in an calcineurin-dependent, but TGF-β- independent manner, comparable to the recently described regulation of fibronectin gene expression in murine renal fibroblasts [46]. Taking these data together, we propose that cadherin-9 is a specific marker for renal fibroblasts: the resident, mature and activated fibroblasts, the cells late in the process of EMT as well as the fibroblastic cells in the CD45+ pool (Fig. 10). However, cadherin-9 is not a general fibroblast marker for all human tissues, since it is not expressed in fibroblast-rich organs such as the skin (Fig. S3).

When human cadherin-9 was originally described, its function was investigated in transfected mouse L-cell clones [29]. In our present study, the interaction of cadherin-9 with catenins was shown *ex vivo* in human kidney cell lysates. Its cellular localization at the cell membrane and the ability to mediate cell-cell interactions were demonstrated in the human kidney fibroblast cell line TK 188, which mimics the natural situation of cadherin-9 in renal fibroblasts more closely than mouse L-cell transfectants. However, only a minor, but significant effect of cadherin-9 on cell-cell aggregation was observed, since knockdown of cadherin-9 decreased the ability to form cell aggregates by less than 15%. This observation might be explained in two ways: Firstly, TK 188 cells express other cell adhesion molecules, which could substitute for the function of cadherin-9. Secondly, an additional function of cadherin-9 seems to be likely, based on its distribution over the entire cell surface and not only concentrated at cell-cell contacts. However, an influence of cadherin-9 on cell migration, as described for mesenchymal cadherins such as N-cadherin [47], could not be found in transmigration and wound healing assays. Thus, one might speculate on a function of cadherin-9 in cell signaling, but strong evidence for such a role is still missing. Shimoyama and coworkers [29] demonstrated *in vitro* the ability of cadherin-9 to mediate not only homophilic, but also heterophilic interactions. This was shown for cadherin-6 and cadherin-10, which are the closest relatives of cadherin-9. Cadherin-10 is not expressed in the adult human kidney (*unpublished data*), but cadherin-6 was found to be expressed in proximal renal tubules. However, double immunofluorescence staining revealed no obvious co-localization of cadherin-6 and –9 at cell contacts between fibroblasts and epithelial cells. Thus, at least in the human kidney, cadherin-9 does not interact with cadherin-6 or cadherin-10 *in vivo*, as they are either not expressed concomitantly (cadherin-10) or not co-localized (cadherin-6).

As cadherin-9 is a specific marker for renal fibroblasts, the question arises whether it might play a role in the process of kidney fibrosis or whether it could be a suitable marker for the disease. In human renal fibrotic tissues, the percentage of cadherin-9-positive cells is clearly elevated. When and how cadherin-9 up-regulation takes place during the course of the disease is difficult to answer, since mice cannot be used as a model for renal cadherin-9 expression in fibrosis. During EMT, cadherin-9 seems to be up-regulated at the later stages. However, whether the higher percentage of cadherin-9-positive cells in renal fibrotic tissue originates from (I) epithelial cells undergoing EMT, (II) from the activation and proliferation of renal fibroblasts, or (III) from kidney infiltrating and differentiating fibrocytes cannot be distinguished yet. According to the cadherin-9 expression pattern, none of these three possibilities of renal fibrosis development can be clearly favored, because our data only show an accumulation of cadherin-9-positive cells in renal fibrosis, and cadherin-9 is expressed by all fibroblast subtypes of the human kidney.

In conclusion, human cadherin-9 is a typical type II cadherin which can mediate homotypic cell-cell interactions. In the kidney, cadherin-9 is a specific marker for renal fibroblasts, but it is not a general marker for fibroblasts in humans. Renal fibroblasts are not a homogeneous cell population, and cadherin-9, together with the marker molecules FSP1, CD45 and α-smooth muscle actin, is an important tool to differentiate renal fibroblasts into fibrocytes, cells undergoing EMT, resident fibroblasts and activated fibroblasts.

## Supporting Information

Figure S1 Cadherin-9 expression is higher in brain than in kidney Cadherin-9 RT-PCR was performed and analyzed after 15, 20, 25, 30 and 35 cycles of amplification. Amplification of β-actin served as an internal control of equal RNA input and quality. The cadherin-9 amplification product appeared first in brain after 20 cycles, in kidney after 25 and in small intestine after 35 cycles. Prostate tissue was negative for cadherin-9. Thus, the amount of cadherin-9 mRNA in the different tissues decreases from brain to kidney to small intestine and is not detectable in prostate. The signal intensities in Fig. 1 seemed to correlate with the expression level of cadherin-9 mRNA in the different tissues.
(2.08 MB TIF)

Figure S2 Cadherin-9 knockdown impairs the ability of renal fibroblasts to aggregate Phase contrast pictures of TK 188 cells treated with cadherin-9 or control siRNA were taken before (0 min) and after 20 min of aggregation. At t = 0 most cells were single cells or very small aggregates (upper row). After 20 min the cadherin-9 siRNA treated cells formed more and smaller aggregates (lower left) compared to the control cells (lower right), showing that the ability of renal fibroblasts to aggregate was impaired by the knockdown of cadherin-9.
(5.27 MB TIF)

Figure S3 Cadherin-9 is not expressed by human skin fibroblasts Immunohistochemical staining of cadherin-9 in human skin cryostat sections revealed no specific staining of any cells compared to the negative control, in which the primary antibody was omitted. Bars represent 50μm. (B) The primary human skin fibroblasts SK 173 were analyzed for cadherin-9 expression by RT-PCR. No cadherin-9 mRNA could be detected. β-actin amplification served as an internal control, and as negative control water instead of RNA was added.
(6.63 MB TIF)
