# Supplementary figures and images for: Cadherin-9 Is a Novel Cell Surface Marker for the Heterogeneous Pool of Renal Fibroblasts
Source: PLoS One. 2007 Aug 1;2(8):e657. doi: 10.1371/journal.pone.0000657 (PMC1924602; doi:10.1371/journal.pone.0000657)

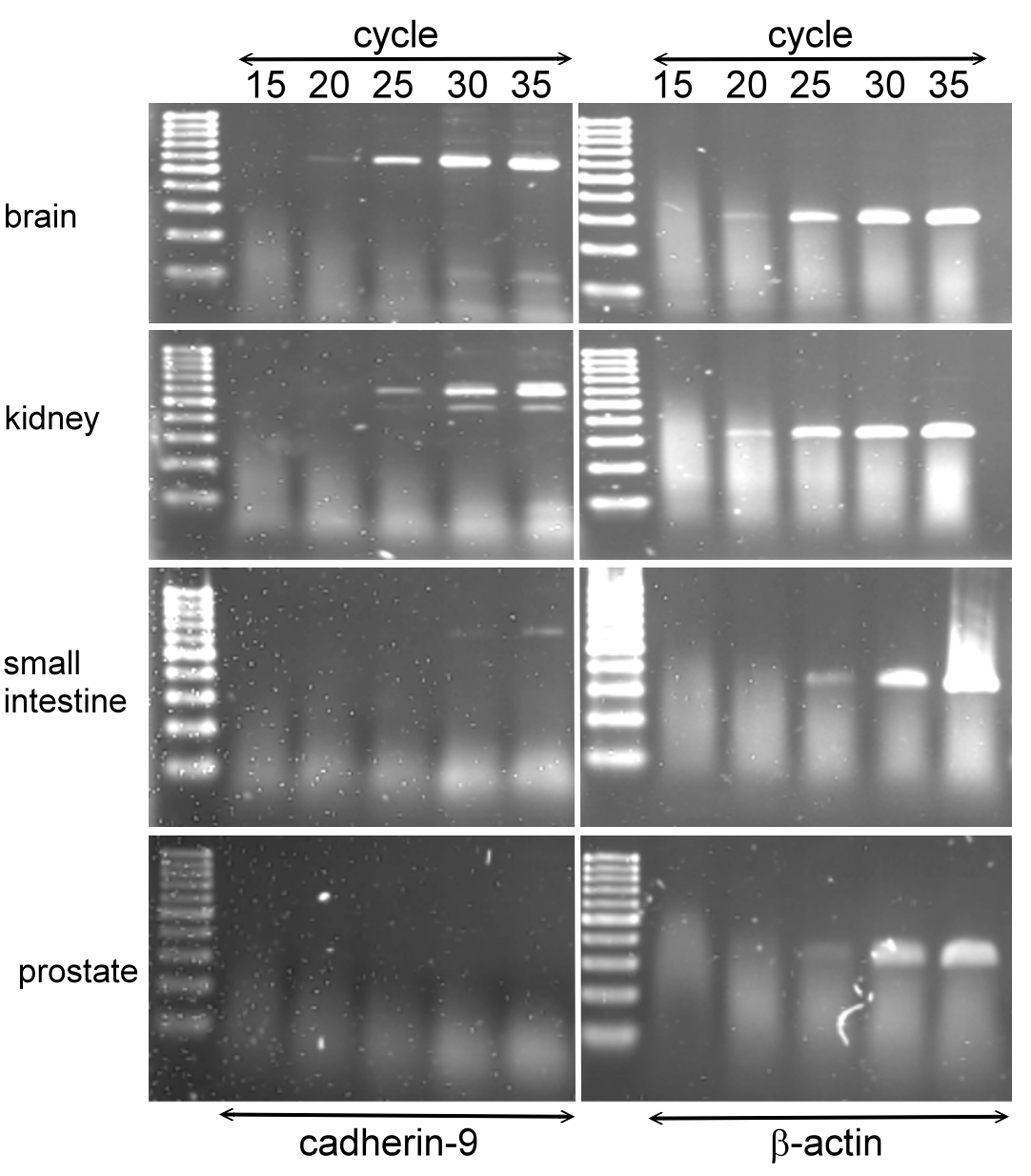

Supplement: Figure S1 — Cadherin-9 expression is higher in brain than in kidney Cadherin-9 RT-PCR was performed and analyzed after 15, 20, 25, 30 and 35 cycles of amplification. Amplification of β-actin served as an internal control of equal RNA input and quality. The cadherin-9 amplification product appeared first in brain after 20 cycles, in kidney after 25 and in small intestine after 35 cycles. Prostate tissue was negative for cadherin-9. Thus, the amount of cadherin-9 mRNA in the different tissues decreases from brain to kidney to small intestine and is not detectable in prostate. The signal intensities in Fig. 1 seemed to correlate with the expression level of cadherin-9 mRNA in the different tissues. (2.08 MB TIF) [file pone.0000657.s001.tif]

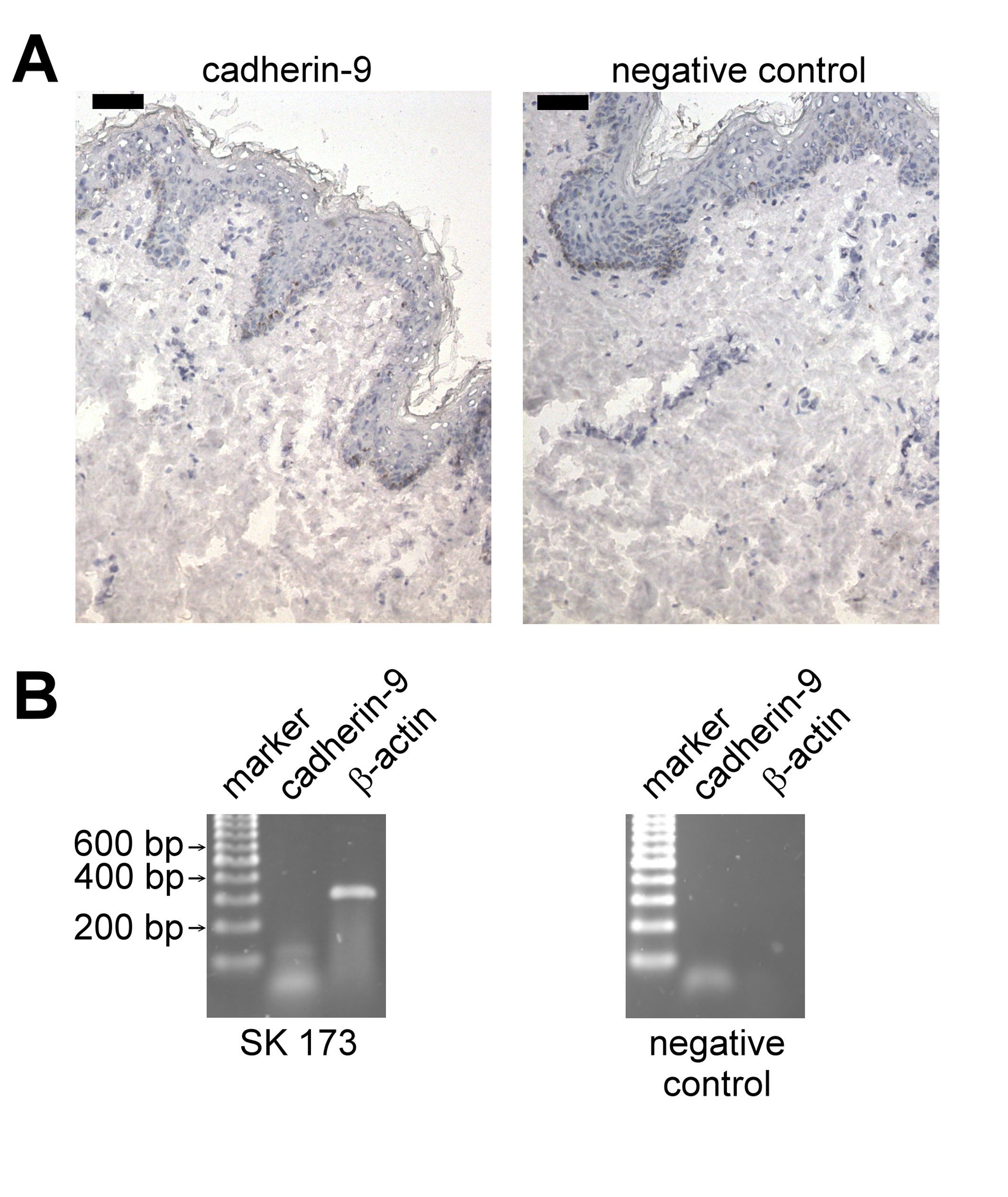

Supplement: Figure S2 — Cadherin-9 knockdown impairs the ability of renal fibroblasts to aggregate Phase contrast pictures of TK 188 cells treated with cadherin-9 or control siRNA were taken before (0 min) and after 20 min of aggregation. At t = 0 most cells were single cells or very small aggregates (upper row). After 20 min the cadherin-9 siRNA treated cells formed more and smaller aggregates (lower left) compared to the control cells (lower right), showing that the ability of renal fibroblasts to aggregate was impaired by the knockdown of cadherin-9. (5.27 MB TIF) [file pone.0000657.s002.tif]

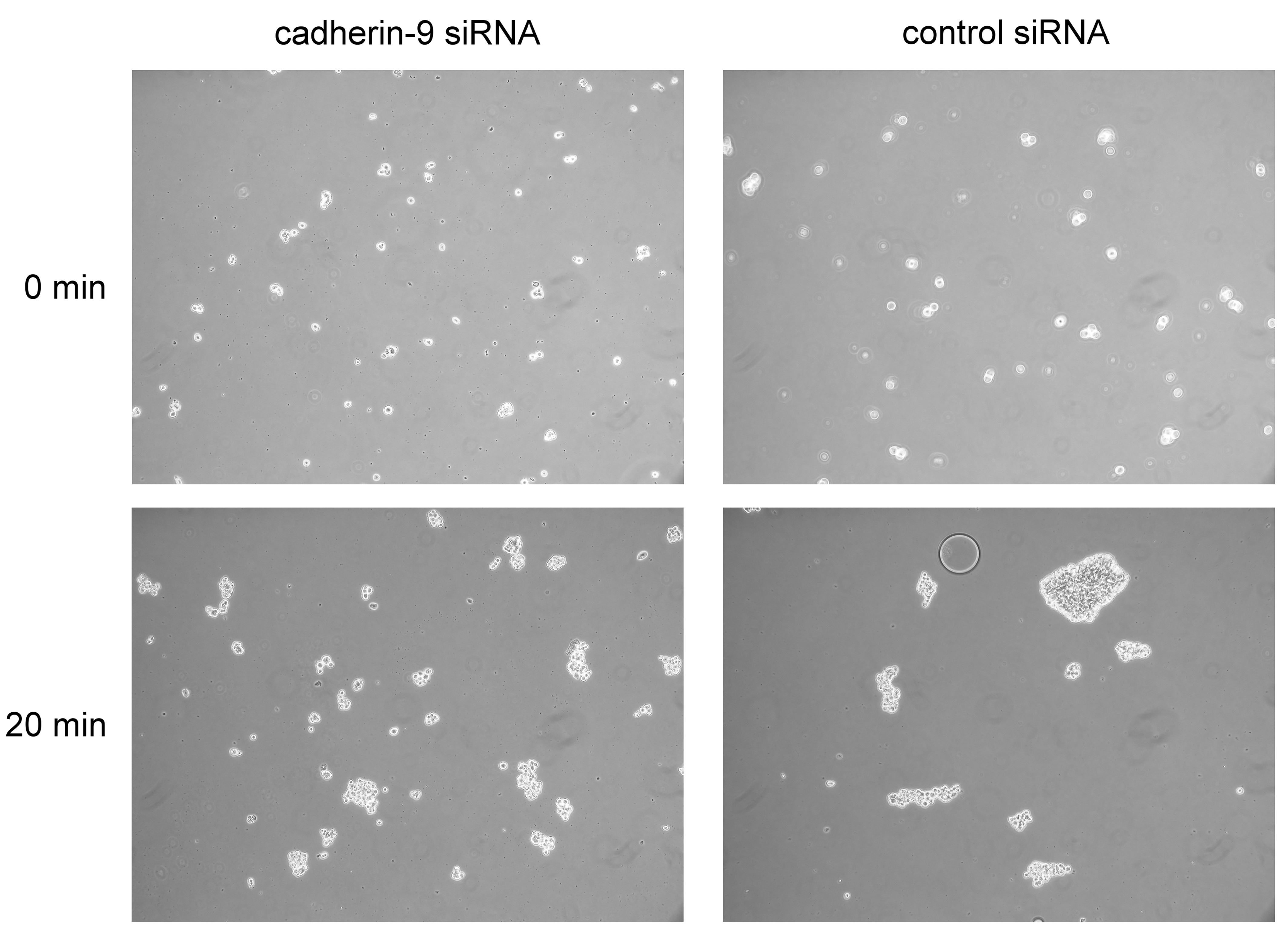

Supplement: Figure S3 — Cadherin-9 is not expressed by human skin fibroblasts Immunohistochemical staining of cadherin-9 in human skin cryostat sections revealed no specific staining of any cells compared to the negative control, in which the primary antibody was omitted. Bars represent 50μm. (B) The primary human skin fibroblasts SK 173 were analyzed for cadherin-9 expression by RT-PCR. No cadherin-9 mRNA could be detected. β-actin amplification served as an internal control, and as negative control water instead of RNA was added. (6.63 MB TIF) [file pone.0000657.s003.tif]
